# Detection and Characterization of the Metabolites of Ciwujianoside B in Rats Based on UPLC-Fusion Lumos Orbitrap Mass Spectrometry

**DOI:** 10.1155/2024/3187511

**Published:** 2024-05-22

**Authors:** Wan-Ru Dong, Xue Gao, Chen-Xue Li, Yan Song, Jun-Hong Chai, Jun Liang

**Affiliations:** Key Laboratory of Basic and Application Research of Beiyao (Heilongjiang University of Chinese Medicine), Ministry of Education, 24 Heping Road, Harbin 150040, China

## Abstract

We previously conducted a systematic study on the metabolic process and products of hederasaponin B in rats. We hypothesized that the sugar chain structures play a key role in the metabolism of triterpenoid saponins. To verify this hypothesis, we conducted metabolic research on ciwujianoside B ascribed to the same sugar chains and a distinct aglycone and compared it with hederasaponin B. Specifically, we collected feces, urine, and plasma of rats after gavage with ciwujianoside B and identified 42 metabolites by UPLC-Fusion Lumos Orbitrap mass spectrometry. Finally, ciwujianoside B metabolism and hederasaponin B metabolism were compared, reaching the following conclusions: (i) more than 40 metabolites were identified in both, with the majority of metabolites identified in feces; (ii) the corresponding metabolic pathways in vivo were basically similar, including deglycosylation, acetylation, hydroxylation, glucuronidation, oxidation, and glycosylation; and (iii) deglycosylation was considered the main metabolic reaction, and its metabolites accounted for approximately 50% of all metabolites. Overall, this study provides a foundation for further research on the metabolism of triterpenoid saponins.

## 1. Introduction

*Acanthopanax senticosus* is a small woody shrub that belongs to the *Araliaceae* family. It is mainly distributed in the northeastern region of China, Korea, and Japan. The rhizome and root of *A. senticosus*, also known as “Siberian ginseng,” have been widely used as a tonic and antifatigue agent for the treatment and prevention of various diseases including cancer, diabetes, ischemic stroke, rheumatism, depression, and Parkinson's disease [1–3]. However, from the perspective of resource utilization, the resources of *A. senticosus* leaves have gradually received attention from the medical and pharmaceutical fields. Pharmacological studies have indicated that *A. senticosus* leaves have multiple bioactivities, e.g., glycosidase inhibition, antiaging, antioxidant, and antitumor effects [4–6]. It has been confirmed that the presence of saponins in the roots, stems, and leaves of *A. senticosus* is responsible for these major effects [7]. However, saponins generally have low bioavailability due to the large chemical polarity and poor oral absorption, and their metabolism has been poorly studied [8, 9]. Therefore, it is essential to elucidate the metabolic fate of triterpene saponins in *A. senticosus* leaves for further exploitation and utilization of its leaf resources.

We previously conducted a systematic study on the metabolic process and products of hederasaponin B, a triterpenoid saponin obtained from *A. senticosus* leaves [10]. We proposed that the sugar chain structures play a key role in the metabolism of triterpenoid saponins. Ciwujianoside B is also a triterpenoid saponin isolated from *A. senticosus* leaves, which has been shown to be able to penetrate and work in the brain, enhance memory function, and confer radioprotective effects [11, 12]. Compared with hederasaponin B, ciwujianoside B is ascribed to the same sugar chains and a distinct aglycone. According to our hypothesis, the metabolism of both should have similar results and searchable rules.

Therefore, in this study, we profiled the in vivo metabolic fate of ciwujianoside B in rats based on the proposed strategy and compared it with that of hederasaponin B. Specifically, we established a UPLC-Fusion Lumos Orbitrap mass spectrometry method for the rapid identification of metabolites of ciwujianoside B in plasma, urine, and feces samples [13, 14]. The metabolite identification results were obtained by using Compound Discoverer 3.0 software combined with manual screening [15, 16]. Subsequently, the possible metabolic pathways of ciwujianoside B were analyzed. Additionally, we compared the results of ciwujianoside B with those of hederasaponin B to summarize the metabolic laws of both. These possible laws could provide valuable reference to further elucidate the metabolism of other similar triterpenoid saponins.

## 2. Materials and Methods

### 2.1. Chemicals and Reagents

*A. senticosus* leaves were collected at Muling County (Heilongjiang Province, China). Mass spectrometry-grade methanol and acetonitrile were obtained from Thermo Fisher (Geel, Belgium). HPLC-grade formic acid was obtained from Dikma (Lake Forest, USA). Purified water was obtained from Watsons (China). Other reagents were purchased from local sources and of analytical grade.

### 2.2. Preparation of Ciwujianoside B

The 3 kg of *A. senticosus* leaves were crushed and extracted with 30 L of 70% ethanol under reflux conditions for three hours. The ethanolic solution was filtered after standing, and extraction procedure was repeated three times. The filtrates were combined and then concentrated by a rotary evaporator. The extract was separated by an AB-8 macroporous resin column (9 cm i.d. ×100 cm) and eluted with water (2.0 BV), 30% ethanol (4.0 BV), 60% ethanol (4.0 BV), and 95% ethanol (4.0 BV). Subsequently, the fraction obtained with 60% ethanol were subjected to silica gel column chromatography (dichloromethane-methanol-water (10 : 1 : 0.1) ⟶ methanol) to obtain six fractions (A–F). Fraction B was subjected to reversed-phase silica gel chromatography (70% methanol-water ⟶ methanol) to obtain three fractions (B_1_–B_3_). Then, fraction B_3_ was purified on a SHIMADZU C18 column (20 × 250 mm, 5 *μ*m) using preparative liquid chromatograph equipped with a refractive index detector. The mobile phase was acetonitrile/water (4 : 6), and the flow rate was 5 mL/min. The retention time of ciwujianoside B was 10.5–10.8 min. Finally, the collected preparation solution was concentrated and freeze-dried to obtain purified ciwujianoside B. Its purity was greater than 98% as determined by HPLC-ELSD.

### 2.3. Animal Experiments

Specific pathogen-free-grade Sprague Dawley male rats (200 ± 20 g) were purchased from the Animal Experiment Center of Heilongjiang University of Chinese Medicine (SYXK (hei) 2021-010). Animals were raised in an environmentally controlled animal room with a temperature of 24 ± 2°C and a 12-h dark/12-h light cycle for a week. And they had free access to water and food during the adaptation period. Then, the rats were randomly divided into three groups (three rats per group): administration group A (collect plasma), administration group B (collect urine and feces), and blank control group C. After fasting for 12 h, groups A and B were given ciwujianoside B dissolved in physiological saline (150 mg/kg) orally, and blank group C was given physiological saline orally. Rats drunk water freely during the experiment. The experimental procedures were approved by the Ethics Committee of Heilongjiang University of Chinese Medicine.

### 2.4. Collection and Preparation of Biosamples

#### 2.4.1. Plasma Samples

Plasma samples were collected from administration groups A. Venous blood samples in the orbit were collected into heparinized tubes at different times (0.25, 0.5, 0.75, 1, 1.5, 2, 3, 4, 6, 8, 10, 12, and 24 h) after oral administration. The blood samples were centrifugated at 3000 rpm. The obtained plasma was transferred and stored at −80°C for further analysis. Before the preparation of biosamples, plasma samples were completely thawed. Each plasma sample (100 *μ*L) was mixed with 700 *μ*L of methanol, swirled for 1 min, and then centrifuged at 12000 rpm for 10 min (4°C). Subsequently, a series of supernatants were merged and dried with nitrogen stream. The residue was redissolved with methanol (100 *μ*L) and centrifuged to obtain the supernatant for analysis. Blank plasma samples were collected and processed as described above.

#### 2.4.2. Feces and Urine Samples

Each rat in groups B was housed in separate metabolic cages, and the feces and urine samples were collected at different time periods (0–4 h, 4–8 h, 8–12 h, 12–24 h, 24–36 h, and 36–48 h) after oral administration. Before the preparation of biosamples, the feces samples were freeze-dried and ground into fine powder. The serial fecal powder samples (0.5 g per serving) were extracted by ultrasound for 30 min using 3 mL of methanol and centrifuged at 12,000 rpm for 10 min (4°C). Then, each supernatant sample (100 *μ*L) was mixed with 700 *μ*L of methanol to precipitate the protein. After centrifugation again, the supernatants were pooled and dried with nitrogen stream. The residue was redissolved with methanol (100 *μ*L) and centrifuged to obtain the supernatant for analysis.

Urine samples were completely thawed at room temperature and then purified by activated SPE cartridges [17]. The purified urine samples (1.0 mL) were combined and dried with nitrogen stream, and the residue was redissolved with methanol (100 *μ*L). After centrifugation and filtration, the supernatant was taken for analysis. Blank feces and urine samples were collected and processed as described above.

### 2.5. Instruments and Conditions

UPLC separation was performed using a Vanquish Flex UPLC system (Thermo Fisher Scientific, USA) on a ACQUITY HSS T3 column (2.1 × 150 mm, 1.8 *μ*m) at a column temperature of 35°C. The mobile phase was water (0.1% formic acid, A) and acetonitrile (0.1% formic acid, B). The UPLC system was eluted with a gradient program as follows: 10–90% B at 0–25 min, 90 − 10% B at 25–25.1 min, 10% B at 25.1–30 min. The flow rate was 0.3 mL/min.

MS analysis was performed using an Orbitrap Fusion Lumos tribrid mass spectrometer equipped with a heating electrospray ionization source (ESI). The following ESI source parameters were used: an ion spray voltage of 3.2 kV, a capillary temperature of 350°C, an ion transfer tube temperature of 320°C, a sheath gas (N_2_) flow rate of 42 arb, a sweep gas (N_2_) flow rate of 1 arb, and an auxiliary gas (N_2_) flow rate of 12 arb. MS spectra were acquired at the mass range of 350–2000 *m*/*z*. High collision-induced dissociation (HCD) was adopted with normalized collision energy setting of 40 eV in ESI^−^ mode and 20 eV in the ESI^+^ mode. MS^2^ spectra were acquired by the data-dependent acquisition (DDA) scan mode, and the primary ions with ionic strength greater than 2.5*e*4 were broken into secondary fragments. Dynamic exclusion was set to 6.00 s.

### 2.6. Data Analysis

The data were recorded in RAW file (.raw) and could be processed using Thermo Scientific Xcalibur 4.2 workstation software. The peaks with intensities above 50,000 were selected for analysis. The Xcalibur files of the blank and administration groups were added into Thermo Scientific Compound Discoverer 3.0 to identify the metabolites of ciwujianoside B, and all data files were analyzed with the same parameter settings. Workflow selected “known compound detection” mode under processing, and the results were exported to a Microsoft Excel spreadsheet. The parameters were set as follows: the degree of unsaturation was 0∼15; the maximum tolerance of mass error was 5 ppm; the elements' composition was C, H, O, N, S, etc.; and other parameters were default values.

## 3. Results and Discussion

### 3.1. Structural Characterization of Ciwujianoside B by NMR

**Figure 1:**
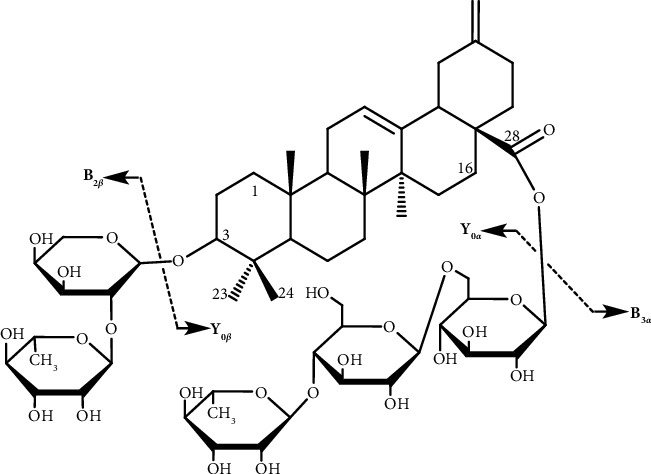
Chemical structure of ciwujianoside B.

The ^1^H-NMR (C_5_D_5_N, 600 MHz) spectrum of ciwujianoside B showed signals for five angular methyls at 1.20, 1.29, 0.85, 1.06, and 1.15 (each, 3H, *s*), two olefinic signals at *δ*_H_ = 5.41 (1 H, *t*, H-12) and 4.66, 4.72 (2H, *br.s*), and five anomeric proton signals suggesting the presence of five sugars (*δ*_H_ = 4.89 (1H, *d*, *J* 4.2 Hz), 5.41 (1H, *d*, *J* 7.0 Hz), 6.19 (1H, *d*, *J* 7.2 Hz), 4.89 (1H, *d*, *J* 7.1 Hz), and 5.84 (1H, *br.s*)). The presence of five sugars in ciwujianoside B was also evident from the five characteristic anomeric carbon signals at *δ*_*C*_ = 104.6, 101.5, 95.5, 104.6, and 102.4 in the ^13^C-NMR (C_5_D_5_N, 150 MHz) spectrum. In addition, the following data provide information on aglycone: two double-bond carbon signals at *δ*_*C*_ = 122.9 and 143.2 (due to C-12 and C-13), a double-bond carbon signal at *δ*_C_ = 107.1 (due to C-29), and a carboxyl carbon signal at *δ*_*C*_ = 175.5 (due to C-28) were observed. Based on the above data, relevant literature [18], and mass spectrometry, we determined that the compound was ciwujianoside B (Figure 1).  Table S1 provides the ^13^C-NMR data and literature comparison for ciwujianoside B.

### 3.2. Structural Characterization of Ciwujianoside B by UPLC-MS^2^ (**M**_0_)

**Figure 2:**
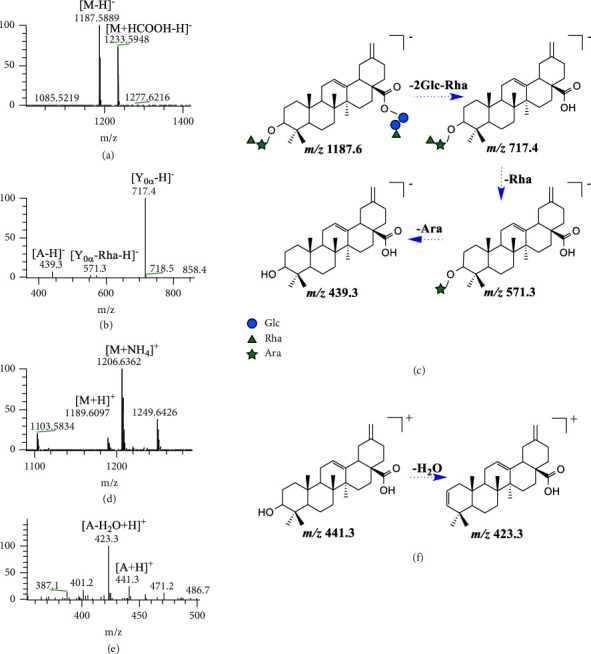
The ESI^−^-MS spectrum (a), ESI^−^-MS/MS spectrum (b), ESI^+^-MS spectrum (d), and ESI^+^-MS^2^ spectrum (e) of **M**_0_ and proposed fragmentation pathways in negative (c) and positive (f) ion modes.

**M**_0_ (C_58_H_92_O_25_, retention time (*t*_*R*_) = 9.86 min) was detected in urine, plasma, and feces samples. Its [M − H]^−^, [M + HCOOH–H]^−^, [M + H]^+^, and [M + NH_4_]^+^ ions were detected at *m*/*z* 1187.5889, 1233.5948, 1189.6097, and 1206.6362, respectively. In the negative ion mode, the fragment ion sequences *m*/*z* 717.4 ⟶ 571.3 ⟶ 553.4 ⟶ 439.3 (Δ*m* = 146, 18, and 114 Da, respectively) were observed in the ESI^−^-MS^2^ spectrum, and [Y_0*α*_ − H]^−^, [Y_0*α*_ − Rha − H]^−^, [Y_0*α*_ − Rha − H_2_OH]^−^, and [A − H]^−^ ions were generated in sequence (A, aglycone). It was speculated that fragmentation of the ester bond was produced at the C-28 position of [M − H]^−^ at *m/z* 1187.6, and then, [Y_0*α*_ − H]^−^ at *m/z* 717.4 continued to break the C-3 glycan chain, thereby showing the above ion peaks. In the positive ion mode, an ion fragment was observed at *m*/*z* 423.3, which could be attributed to [A − H_2_O + H]^+^, and the aglycone was assumed to be akebonoic acid [19]. According to the above evidences, it can be inferred that the main structure of **M**_0_ was Rha ⟶ Ara-A-Glc ⟵ Glc ⟵ Rha. The main cleavage characteristics are shown in Figure 2, which helped to identify other metabolites in vivo.

### 3.3. Structural Characterization of Ciwujianoside B Metabolites

**Figure 3:**
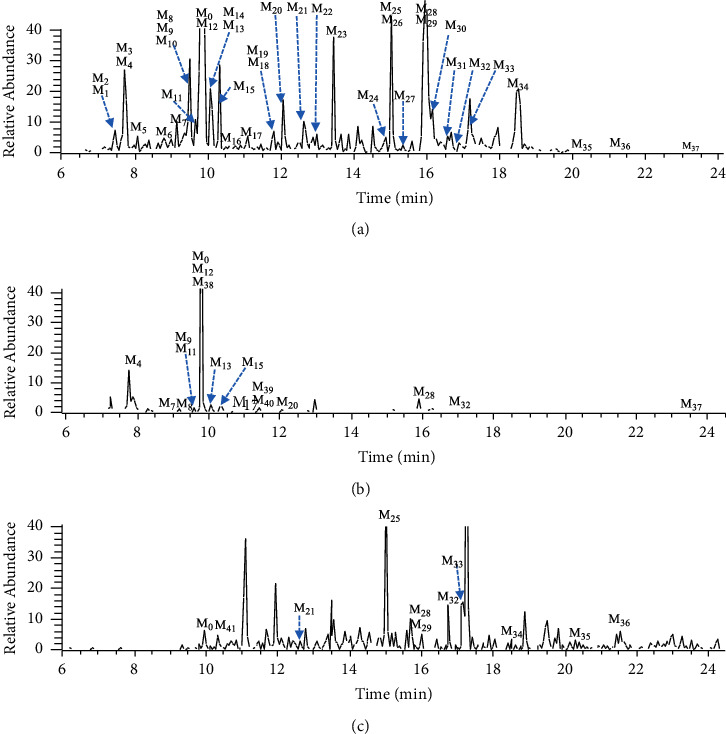
UPLC-MS extracted ion chromatograms (EICs) of the metabolites in rat feces (a), urine (b), and plasma (c) of ciwujianoside B in the negative ion mode.

By analyzing the mass detection results of the treated biological samples and the corresponding blank samples, 42 metabolites (**M**_0_–**M**_41_) were preliminarily identified (Figure 3). The corresponding metabolic pathways were proposed, including deglycosylation, acetylation, hydroxylation, oxidation, glycosylation, and glucuronidation reactions.  Table 1 provides the detailed UPLC-MS^2^ data of ciwujianoside B metabolites. In the following text, typical examples of different metabolic pathways are discussed in detail.

#### 3.3.1. Deglycosylated Metabolites (**M**_12_, **M**_14_, **M**_15_, **M**_18_, **M**_19_, **M**_21_, **M**_26_, **M**_28_, **M**_30_, **M**_29_, **M**_33_, **M**_34_, and **M**_36_)

Deglycosylation is the main metabolic pathway of ciwujianoside B. A total of 13 single deglycosylated metabolites were identified (**M**_12_, **M**_14_, **M**_15_, **M**_18_, **M**_19_, **M**_21_, **M**_26_, **M**_28_, **M**_30_, **M**_29_, **M**_33_, **M**_34_, **M**_36_), four of which (**M**_12_, **M**_18_, **M**_28_, and **M**_30_) are important products of single deglycosylation reaction, and they are discussed below as typical examples.

**M**_12_, **M**_18_, **M**_28_, and **M**_30_ (*t*_*R*_ = 9.86, 11.76, 15.90, and 16.25 min, respectively) are isomers produced through rearrangements of the sugar chain and glycosyl isomerization. Their molecular formula is C_40_H_62_O_11_, and their molecular weight is 718 Da, which is 470 Da less than that of M_0_. The oligosaccharide chains of the prototype drug are prone to break from the outside to the inside, resulting in the metabolites that lose different glycosyls. This can manifest as a difference in the molecular weight—132, 146, and 162 Da—corresponding to the loss of arabinose (Ara), rhamnose (Rha), and glucose (Glc). Thus, it could be speculated that **M**_12_, **M**_18_, **M**_28_, and **M**_30_ are generated through the removal of the C-28 sugar chain (Glc ← Glc ← Rha) during the metabolic process of the prototype drug. **M**_12_ produced [M − H]^−^ ions at *m*/*z* 717.4255 and [M + HCOOH–H]^−^ ions at *m*/*z* 763.4311 in the ESI^−^-MS spectrum. As shown in Figure S1, rhamnose (162 Da) was first lost in the C-3 sugar chain of [M − H]^−^, which resulted in a group of fragment ions [M − Rha − H]^−^ at *m*/*z* 571.4, [M − Rha − H_2_O – H]^−^ at *m*/*z* 553.4, and [A − H]^−^ at *m*/*z* 439.3. The structure of **M**_12_, **M**_18_, **M**_28_, and **M**_30_ was preliminarily identified as Rha ⟶ Ara-A. The specific mass spectrum and cleavage pathways are shown in Figure S1.

#### 3.3.2. Deglycosylated and Hydroxylated Metabolites (**M**_2_, **M**_5_, **M**_10_, **M**_20_, **M**_22_, **M**_24_, **M**_25_, **M**_27_, **M**_32_, **M**_35_, **M**_38_, and **M**_39_)

**Figure 4:**
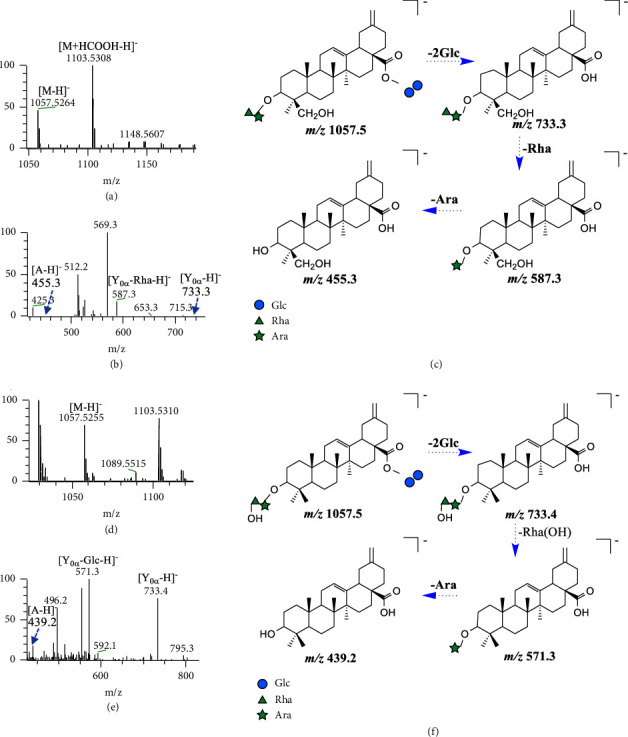
The ESI^−^-MS spectrum (a) and ESI^−^-MS/MS spectrum (b) of **M**_5_ and proposed fragmentation pathway (c). The ESI^−^-MS spectrum (d) and ESI^−^-MS/MS spectrum (e) of **M**_10_ and proposed fragmentation pathway (f).

**M**_5_, **M**_10_, and **M**_39_ (*t*_*R*_ = 8.01, 9.56, 11.31 min, respectively) are analyzed below as representative metabolites. Their molecular formula is C_52_H_82_O_22_, and their molecular weight is 1058 Da. In the negative ion mode, [M − H]^−^ at *m*/*z* 1057.5264 and [M + HCOOH–H]^−^ at *m*/*z* 1103.5308 of **M**_5_ were observed in the MS spectrum. When the collision energy HCD was 40 eV, the ion fragmentation sequence of **M**_5_ was *m*/*z* 733.3 ⟶ 587.3 ⟶ 569.3 ⟶ 455.3 (Δm = 146, 18, and 114 Da in the sequence), and [Y_0*α*_ − H]^−^, [Y_0*α*_ − Rha − H]^−^, [Y_0*α*_ − Rha − H_2_OH]^−^, and [A − H]^−^ ion fragments were generated in turn. This shows that the hydroxylation of **M**_5_ occurs on aglycone. The fragment ion information of **M**_39_ was similar to that of **M**_5_. Another metabolite **M**_10_ showed the ion fragmentation sequence *m*/*z* 733.4 ⟶ 571.3 ⟶ 553.2 ⟶ 439.2 (Δm = 162, 18, and 114 Da in the sequence), and [Y_0*α*_ − H]^−^, [Y_0*α*_−Rha (OH)–H]^−^, [Y_0*α*_−Rha (OH)–H_2_O–H]^−^, and [A−H]^−^ ion fragments were generated in turn. This indicates that the hydroxylation of **M**_10_ occurs at the C-3 position sugar chain. According to the above evidences, **M**_5_, **M**_10_, and **M**_39_ were preliminarily identified as Rha ⟶ Ara-A (OH)-Glc ⟵ Glc or Rha (OH) ⟶ Ara-A-Glc ⟵ Glc. The specific mass spectrum and cleavage pathways are shown in Figure 4.

#### 3.3.3. Deglycosylated and Acetylated Metabolites (**M**_17_, **M**_23_, and **M**_31_)

**M**_17_, **M**_23_, and **M**_31_ are generated through the acetylation and deglycosylation of **M**_0_. Here, **M**_23_ (*t*_*R*_ = 13.66 min) is taken as an example. Its molecular structural formula is C_49_H_77_O_18_, and the molecular weight is 952 Da, which is 236 Da less than that of M_0_. It could be speculated that **M**_23_ was generated through the removal of Rha ⟶ Ara at the C-3 position and the addition of an acetyl group during the metabolic process of the prototype drug. The ESI^−^-MS spectrum of **M**_23_ showed the fragment ions of [M − H]^−^ at *m*/*z* 951.4993 and [M − HCOOH–H]^−^ at *m*/*z* 997.5049. The MS^2^ spectrum of **M**_23_ showed the fragment ions at *m/z* 439.4 ([A − H]^−^), which reflected aglycone information. These data indicate that **M**_23_ is produced by acetylation at a certain location in the C-28 sugar chain after the removal of the C-3 sugar chain (Rha ⟶ Ara) of the prototype drug, or by the acetylation of the metabolite **M**_21_. Therefore, **M**_23_ was preliminarily identified as A-Glc ⟵ Glc ⟵ Rha (Ac) (Figure S2).

#### 3.3.4. Oxidated and Deglycosylated Metabolite (**M**_37_)

The molecular formula of **M**_37_ (*t*_*R*_ = 23.65 min) is C_29_H_42_O_5_. The ESI ^+^ -MS^2^ spectrum showed the fragment ion of [A − H_2_O + H]^+^ located at *m*/*z* 453.2, which provided aglycone information. Therefore, it could be speculated that **M**_37_ is produced by both hydroxylation and oxidation on the aglycone, as shown in Figure S3.

#### 3.3.5. Hydroxylated Metabolites (**M**_1_, **M**_3_, **M**_4_, **M**_6_, and **M**_41_)

**M**_1_, **M**_3_, **M**_4_, **M**_6_, and **M**_41_ are hydroxylation products of **M**_0_. Among them, **M**_1_ and **M**_3_ are isomers of each other, and they have the same molecular formula (C_58_H_92_O_27_). The molecular weight of **M**_1_ and **M**_3_ is 32 Da higher than that of **M**_0_, so it can be speculated that **M**_0_ undergoes two hydroxylation reactions to produce **M**_1_ and **M**_3_. According to the detailed MS^2^ data of **M**_1_ and **M**_3_ (Table 1), it can be assumed that the two hydroxylation reactions of **M**_1_ occur one on aglycone and one on rhamnose at C-3, while both reactions of **M**_3_ occur on aglycone.

**M**_4_, **M**_6_, and **M**_41_ have the same molecular formula (C_58_H_92_O_26_). According to Table 1, the ion fragmentation sequence of **M**_4_ and **M**_41_ was *m*/*z* 1203.6 ⟶ 733.4 ⟶ 587.2 ⟶ 455.3 (Δ*m* = 470, 146, and 132 Da), and [M − H]^−^, [Y_0*α*_ − H]^−^, [Y_0*α*_ − Rha − H]^−^, and [A − H]^−^ ion fragments were generated in turn. It indicates that the hydroxylation of **M**_4_ and **M**_41_ occurs on aglycones. As for **M**_6_, the fragment sequence was *m*/*z* 1203.6 ⟶ 733.3 ⟶ 571.3 ⟶ 439.4 (Δ*m* = 470, 162, and 132 Da), and [M − H]^−^, [Y_0*α*_−H]^−^, [Y_0*α*_ − Rha (OH)–H]^−^, and [A − H]^−^ ion fragments were generated in turn. It could be speculated that the hydroxylation of **M**_6_ occurs on rhamnose at C-3. Based on the above data, the structures of **M**_4_, **M**_41_, and **M**_6_ were speculated to be Rha ⟶ Ara-A (OH)-Glc ⟵ Glc ⟵ Rha and Rha (OH) ⟶ Ara-A-Glc ← Glc ← Rha (Figure S4).

#### 3.3.6. Acetylated Metabolite (**M**_16_)

**Figure 5:**
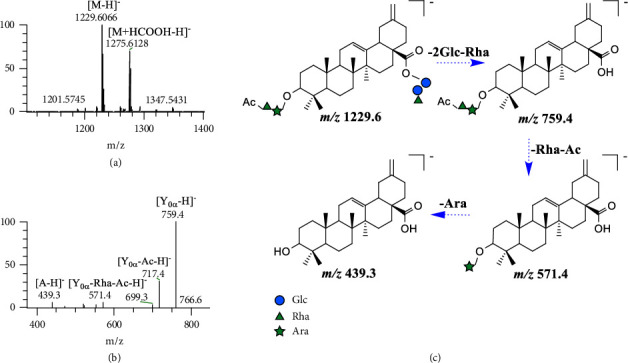
The ESI^−^-MS spectrum (a) and ESI^−^-MS/MS spectrum (b) of **M**_16_ and proposed fragmentation pathway (c).

[M − H]^−^ ions of **M**_16_ (*t*_*R*_ = 10.56 min, C_60_H_94_O_26_) were detected at *m*/*z* 1229.6066. According to [M − H]^−^ ⟶ [Y_0*α*_ − H]^−^ (Δ*m* = 470), it could be speculated that [Y_0*α*_ − H]^−^ is formed by the loss of “Glc ⟵ Glc ⟵ Rha.” The fragment ion sequence was observed as follows: [Y_0*α*_ − H]^−^ (*m*/*z* 759.4) ⟶ [Y_0*α*_ − Ac − H]^−^ (*m*/*z* 717.4) ⟶ [Y_0*α*_ − Rha − Ac − H]^−^ (*m*/*z* 571.4) ⟶ [A − H]^−^ (*m*/*z* 439.3). It was preliminarily determined that the structure of **M**_16_ is (Ac) Rha ⟶ Ara − A − Glc ⟵ Glc ⟵ Rha (as shown in Figure 5).

#### 3.3.7. Hydroxylated and Acetylated Metabolites (**M**_13_ and **M**_40_)

**M**_13_ (*t*_*R*_ = 10.06 min, C_60_H_94_O_27_) and **M**_40_ (*t*_*R*_ = 11.45 min, C_60_H_94_O_27_) are the products of simultaneous acetylation and hydroxylation of the parent drug, which generated fragment ions of [M − H]^−^ located at *m*/*z* 1245.5961. When the collision energy HCD was 40 eV, the characteristic fragment ion sequences *m*/*z* 775.5 ⟶ 733.4 ⟶ 571.3 ⟶ 439.3 (Δ*m* = 42, 162, and 132 Da) were observed in the ESI^−^-MS^2^ spectrum, and [Y_0*α*_ − H]^−^, [Y_0*α*_ − Ac − H]^−^, [Y_0*α*_ − Glc − Ac − H]^−^, and [A − H]^−^ ions were generated in sequence. The ESI^+^-MS^2^ spectrum showed an [A − H_2_O + H]^+^ ion, which provided aglycone information at *m*/*z* 423.4. Presumably, the acetyl group of **M**_13_ is added to the end of the oligosaccharide chain at C-3, while hydroxylation of rhamnose occurs at the C-3 position compared with **M**_0_. Therefore, the structure of **M**_13_ may be (Ac) Rha (OH) ⟶ Ara − A − Glc ← Glc ← Rha. In addition, the MS^2^ fragment of **M**_40_ occurred at *m*/*z* 733.6 ⟶ 587.4 ⟶ 455.3 (Δ*m* = 146 and 132 Da), so it is uncertain whether the acetylation reaction occurs on the C-3 or the C-28 position sugar chain, but the hydroxylation reaction occurs on the aglycone. Thus, it could be speculated that the structure of **M**_40_ is (Ac) Rha ⟶ Ara − A (OH) − Glc ← Glc ← Rha (Figure S5).

#### 3.3.8. Glycosylated Metabolites (**M**_9_, **M**_11_)

This section provides the data of the glycosylation metabolite **M**_9_. The formula of **M**_9_ (*t*_*R*_ = 9.51 min) is C_64_H_102_O_30_, and its molecular weight is 162 Da greater than that of **M**_0_. It may be speculated that **M**_9_ has an additional glucose group. In the negative ion mode, the [M − H]^−^ ion of **M**_9_ was detected at *m*/*z* 1349.6433. In the ESI^−^-MS^2^ spectrum, the characteristic fragmentation ions sequence was *m*/*z* 879.5 ([Y_0*α*_ − H]^−^) ⟶ *m*/*z* 717.4 ([Y_0*α*_ − Glc − H]^−^) ⟶ *m*/*z* 571.3 ([Y_0*α*_ − Glc − Rha − H]^−^) ⟶ *m*/*z* 439.3 ([A − H]^−^) (Δ*m* = 162, 146, and 132 Da in the sequence). This indicates that the sugar chain structure (C-3) of the metabolite is Glc ⟶ Rha ⟶ Ara. Based on these data, it was preliminarily determined that **M**_9_ is Glc ⟶ Rha ⟶ Ara-A-Glc ⟵ Glc ⟵ Rha (Figure S6). Similarly, the formula of **M**_11_ (*t*_*R*_ = 10.03 min) is C_63_H_100_O_29_, and its molecular weight is 132 Da greater than that of **M**_0_. It may be speculated that **M**_9_ has an additional arabinose group. In the negative ion mode, [M − H]^−^ ion of **M**_11_ was detected at *m/z* 1319.6329. The MS^2^ fragment of **M**_11_ occurred at *m/z* 849.4 ([Y_0*α*_-H]^−^), 717.4 ([Y_0*α*_-Ara-H]^−^), 571.3 ([Y_0*α*_-Ara-Rha-H]^−^), 553.3 ([Y_0*α*_-Ara-Rha-H_2_O-H]^−^), and 439.3 ([A-H]^−^). Accordingly, the structure of the C-3 sugar chain is inferred to be Ara ⟶ Rha ⟶ Ara. Based on the above evidences, **M**_11_ was preliminarily determined as Ara ⟶ Rha ⟶ Ara-A-Glc ⟵ Glc ⟵ Rha (Figure S6).

#### 3.3.9. Glucuronidated Metabolites (**M**_7_, **M**_8_)

The molecular weight of **M**_7_ (*t*_*R*_ = 9.16 min) and **M**_8_ (*t*_*R*_ = 9.46 min) is 1364 Da, which is 176 Da greater than that of the prototype drug. It may be speculated that **M**_7_ and **M**_8_ are the metabolites of glucuronidation of **M**_0_. The ESI^−^-MS^2^ spectrum of **M**_7_ and **M**_8_ showed characteristic fragmentation ions sequence *m*/*z* 893.5 ([Y_0*α*_ − H]^−^) ⟶ *m*/*z* 717.4 ([Y_0*α*_−GlcA−H]^−^) ⟶ *m*/*z* 571.3 ([Y_0*α*_−GlcA−Rha−H]^−^) ⟶ *m*/*z* 439.3 ([A−H]^−^) (Δ*m* = 176, 146, and 114 Da in the sequence). Moreover, the [Y_0*α*_−H]^−^ ion at *m*/*z* 893.5 strongly suggests that **M**_7_ and **M**_8_ have extra glucuronic acid groups in the C-3 position sugar chain. Therefore, it was preliminarily determined that the structure of **M**_7_ is GlcA ⟶ Rha ⟶ Ara-A-Glc ← Glc ← Rha (Figure S7).

### 3.4. Metabolic Pathways of Ciwujianoside B

A total of 42 metabolites were tentatively identified, including 38 metabolites in feces, 17 metabolites in urine, and 11 metabolites in plasma. It is noticeable that **M**_0_, **M**_28_, and **M**_32_ were simultaneously found in rat plasma, urine, and feces. A few metabolites were found only in urine (i.e., **M**_38–40_) or plasma (i.e., **M**_41_), and most of them were detected in fecal samples (i.e., **M**_0–37_) (Figure S8). It may be speculated that the main excretion route of ciwujianoside B was through feces. Deglycosylation products **M**_30_ and **M**_33_ were the most abundant components found in rat feces and plasma, indicating that deglycosylation is an important metabolic reaction of ciwujianoside B. Other metabolic pathways were similar to those of hederasaponin B, including acetylation, hydroxylation, glucuronidation, oxidation, and glycosylation.

**Figure 6:**
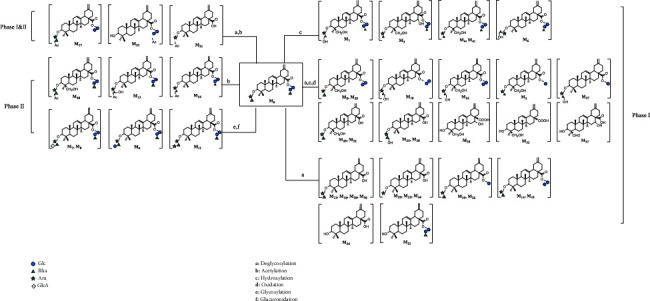
Proposed metabolic pathways of ciwujianoside B in rats.

Due to the similar metabolic pathway, it may also be speculated that the deglycosylation of ciwujianoside B was likely to be influenced by the gut microbiota to produce a series of more easily absorbable secondary glycosides, and then further hydroxylation and redox reactions through CYP 450 to product more metabolites, which are eventually discharged out of the body. Among them, there are 31 metabolites of phase I, seven metabolites of phase II, and three metabolites involved in both phase I and phase II metabolism. The classification of all metabolites and possible metabolic pathways is shown in Figure 6.

**Figure 7:**
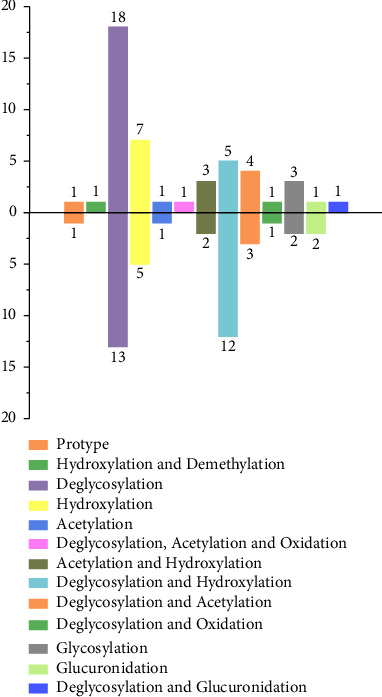
The numbers of metabolites of hederasaponin B (up direction) and ciwujianoside B (down direction) by different metabolic pathways.

On the basis of our previous research on the metabolism of hederasaponin B in vivo, some interesting points could be found by comparison. Firstly, more than 40 metabolites were found in both studies, with the majority found in feces. Secondly, as shown in Figure 7, the metabolic pathways of hederasaponin B and ciwujianoside B were basically similar, including phase I reactions such as deglycosylation, hydroxylation, demethylation, and oxidation, phase II reactions such as mainly acetylation, glycosylation, and glucuronidation, and the number of metabolites produced by different metabolic pathways was also basically the same. Thirdly, deglycosylated metabolites account for approximately 50% of all metabolites, which implies that deglycosylation was the main metabolic pathway of both. Presumably, due to the poor absorption of saponins after oral administration, they undergo deglycosylation in the gut microbiota to produce secondary glycosides for better absorption [20, 21]. Under the action of CYP 450, further reactions such as hydroxylation and redox occur. In addition, by analyzing the cleavage behaviors of products and different metabolic pathways, it was found that the deglycosylation reaction mainly removes the C-28 sugar chain. Glycosylation and glucuronidation mainly occurred at the C-3 sugar chain, while hydroxylation tended to occur on the rhamnosyl and aglycone.

## 4. Conclusion

The metabolism of ciwujianoside B in vivo was systematically studied for the first time, and the main research results are as follows. The metabolic pathways of ciwujianoside B involve deglycosylation, acetylation, hydroxylation, glucuronidation, oxidation, and glycosylation reactions. Deglycosylation was considered the main metabolic reaction. A total of 42 metabolites (**M**_0_–**M**_41_) were preliminarily identified, and 38, 17, and 11 metabolites were found in feces, urine, and plasma. They include 31 phase I metabolites and seven phase II metabolites, and three products are involved in both phase I and phase II metabolism. In addition, ciwujianoside B metabolism and hederasaponin B metabolism were compared, which confirmed our hypothesis. In short, this study systematically explored the metabolic fate of ciwujianoside B and provided a valuable reference for elucidating the postadministration metabolism of other triterpene saponins.

## Figures and Tables

**Table 1:** Metabolites identified in rat biological samples by UPLC-Fusion Lumos Orbitrap mass spectrometer.

No	Formula	[M-H]^−^	*t*_*R*_	Fragmentation ions (intensity %) of [M-H]^−^/[M+H]^+^	Transformation
**M**_0_	C_58_H_92_O_25_	1187.5889	9.86	717.4 (100%) [Y_0*α*_-H]^−^, 571.3 (8%) [Y_0*α*_-Rha-H]^−^, 553.4 (7%) [Y_0*α*_-Rha-H_2_O-H]^−^, 439.3 (12%) [A-H]^−^423.3 (50%) [A-H_2_O + H]^+^	P
**M**_1_	C_58_H_92_O_27_	1219.5820	7.40	749.5 (20%) [Y_0*α*_-H]^−^, 587.4 (5%) [Y_0*α*_-Rha (OH)–H]^−^, 455.3 (50%) [A-H]^−^439.4 (20%) [A-H_2_O + H]^+^	H
**M**_2_	C_46_H_72_O_17_	895.4731	7.50	733.4 (60%) [Y_0*α*_-H]^−^, 587.3 (60%) [Y_0*α*_-Rha-H]^−^, 455.3 (100%) [A-H]^−^	D + H
**M**_3_	C_58_H_92_O_27_	1219.5809	7.68	749.4 (100%) [Y_0*α*_-H]^−^, 603.3 (10%) [Y_0*α*_-Rha-H]^−^, 585.3 (10%) [Y_0*α*_-Rha-H_2_O-H]^−^, 471.2 (2%) [A-H]^−^	H
**M**_4_	C_58_H_92_O_26_	1203.5853	7.71	733.4 (50%) [Y_0*α*_-H]^−^, 587.2 (10%) [Y_0*α*_-Rha-H]^−^, 569.3 (12%) [Y_0*α*_-Rha-H_2_O-H]^−^, 455.3 (10%) [A-H]^−^439.3 (60%) [A-H_2_O + H]^+^	H
**M**_5_	C_52_H_82_O_22_	1057.5264	8.01	733.3 (2%) [Y_0*α*_-H]^−^, 587.3 (20%) [Y_0*α*_-Rha-H]^−^, 569.3 (100%) [Y_0*α*_-Rha-H_2_O-H]^−^, 455.3 (2%) [A-H]^−^439.2 (30%) [A-H_2_O + H]^+^	D + H
**M**_6_	C_58_H_92_O_26_	1203.5839	9.01	733.3 (20%) [Y_0*α*_-H]^−^, 571.3 (70%) [Y_0*α*_-Rha (OH)–H]^−^, 553.3 (40%) [Y_0*α*_-Rha (OH)–H_2_O–H]^−^, 439.4 (90%) [A-H]^−^423.3 (100%) [A-H_2_O + H]^+^	H
**M**_7_	C_64_H_100_O_31_	1363.6216	9.16	893.5 (100%) [Y_0*α*_-H]^−^, 717.4 (60%) [Y_0*α*_-GlcA-H]^−^, 571.3 (50%) [Y_0*α*_-GlcA-Rha-H]^−^, 553.3 (30%) [Y_0*α*_-GlcA-Rha-H_2_OH]^−^, 439.3 (30%) [A-H]^−^423.3 (85%) [A-H_2_O + H]^+^	Gu
**M**_8_	C_64_H_100_O_31_	1363.6271	9.46	893.5 (100%) [Y_0*α*_-H]^−^, 717.4 (70%) [Y_0*α*_-GlcA-H]^−^, 571.4 (40%) [Y_0*α*_-GlcA-Rha-H]^−^, 553.3 (30%) [Y_0*α*_-GlcA-Rha-H_2_OH]^−^, 439.3 (50%) [A-H]^−^	Gu
**M**_9_	C_64_H_102_O_30_	1349.6433	9.51	879.5 (5%) [Y_0*α*_-H]^−^, 717.4 (100%) [Y_0*α*_-Glc-H]^−^, 571.3 (30%) [Y_0*α*_-Glc-Rha-H]^−^, 553.4 (20%) [Y_0*α*_-Glc-Rha-H_2_O-H]^−^, 439.3 (60%) [A-H]^−^423.4 (80%) [A-H_2_O + H]^+^	G
**M**_10_	C_52_H_82_O_22_	1057.5255	9.56	733.4 (80%) [Y_0*α*_-H]^−^, 717.3 (15%) [Y_0*α*_-H_2_O–H]^−^, 571.3 (100%) [Y_0*α*_-Glc-H]^−^, 553.2 (90%) [Y_0*α*_-Glc-H_2_O-H]^−^, 439.2 (20%) [A-H]^−^	D + H
**M**_11_	C_63_H_100_O_29_	1319.6329	9.66	849.4 (10%) [Y_0*α*_-H]^−^, 717.4 (80%) [Y_0*α*_-Ara-H]^−^, 571.3 (60%) [Y_0*α*_-Ara-Rha-H]^−^, 553.3 (40%) [Y_0*α*_-Ara-Rha-H_2_O-H]^−^, 439.3 (100%) [A-H]^−^	G
**M**_12_	C_40_H_62_O_11_	717.4255	9.86	571.4 (10%) [M-Rha-H]^−^, 553.4 (10%) [M-Rha-H_2_O-H]^−^, 439.3 (20%) [A-H]^−^423.3 (100%) [A-H_2_O + H]^+^	D
**M**_13_	C_60_H_94_O_27_	1245.5961	10.06	775.5 (30%) [Y_0*α*_-H]^−^, 733.4 (80%) [Y_0*α*_-Ac-H]^−^, 571.3 (100%) [Y_0*α*_-Glc-Ac-H]^−^, 553.3 (40%) [Y_0*α*_-Glc-Ac-H_2_O-H]^−^, 439.3 (25%) [A-H]^−^423.4 (80%) [A-H_2_O + H]^+^	H + Ac
**M**_14_	C_52_H_82_O_21_	1041.5306	10.11	717.4 (100%) [Y_0*α*_-H]^−^, 571.4 (5%) [Y_0*α*_-Rha-H]^−^, 553.4 (5%) [Y_0*α*_-Rha-H_2_O-H]^−^, 439.4 (10%) [A-H]^−^423.3 (100%) [A-H_2_O + H]^+^	D
**M**_15_	C_52_H_82_O_21_	1041.5350	10.31	717.4 (10%) [Y_0*α*_-H]^−^, 571.3 (100%) [Y_0*α*_-Rha-H]^−^, 553.3 (1%) [Y_0*α*_-Rha-H_2_O-H]^−^, 439.2 (2%) [A-H]^−^423.3 (100%) [A-H_2_O + H]^+^	D
**M**_16_	C_60_H_94_O_26_	1229.6066	10.56	759.4 (100%) [Y_0*α*_-H]^−^, 717.4 (35%) [Y_0*α*_-Ac-H]^−^, 571.4 (6%) [Y_0*α*_-Rha-Ac-H]^−^, 553.4 (5%) [Y_0*α*_-Rha-Ac-H_2_O-H]^−^, 439.3 (6%) [A-H]^−^423.3 (40%) [A-H_2_O + H]^+^	Ac
**M**_17_	C_54_H_84_O_22_	1083.5410	11.11	759.4 (10%) [Y_0*α*_-H]^−^, 717.4 (50%) [Y_0*α*_-Ac-H]^−^, 571.3 (100%) [Y_0*α*_-Rha-Ac-H]^−^, 439.3 (10%) [A-H]^−^423.3 (60%) [A-H_2_O + H]^+^	D + Ac
**M**_18_	C_40_H_62_O_11_	717.4254	11.76	571.4 (10%) [M-Rha-H]^−^, 553.3 (10%) [M-Rha-H_2_O-H]^−^, 439.3 (20%) [A-H]^−^	D
**M**_19_	C_46_H_72_O_16_	879.4787	11.81	717.4 (100%) [Y_0*α*_-H]^−^, 571.3 (5%) [Y_0*α*_-Rha-H]^−^, 553, 3 (5%) [Y_0*α*_-Rha-H_2_O-H]^−^, 439.3 (8%) [A-H]^−^423.3 (15%) [A-H_2_O + H]^+^	D
**M**_20_	C_40_H_62_O_12_	733.4202	12.11	587.3 (100%) [M-Rha-H]^−^, 569.3 (20%) [M-Rha-H_2_O-H]^−^, 455.3 (40%) [A-H]^−^	D + H
**M**_21_	C_47_H_74_O_17_	909.4930	12.61	439.3 (100%) [A-H]^−^423.3 (100%) [A-H_2_O + H]^+^	D
**M**_22_	C_40_H_62_O_12_	733.4217	13.16	717.3 (2%) [M-OH-H]^−^, 587.3 (20%) [M-Rha-H]^−^, 569.3 (20%) [M-Rha-H_2_O-H]^−^, 455.3 (30%) [A-H]^−^	D + H
**M**_23_	C_49_H_76_O_18_	951.4993	13.66	439.4 (100%) [A-H]^−^423.4 (40%) [A-H_2_O + H]^+^	D + Ac
**M**_24_	C_52_H_82_O_23_	1073.5267	14.81	749.5 (20%) [Y_0*α*_-H]^−^, 587.4 (40%) [Y_0*α*_-Rha (OH)–H]^−^, 569.3 (20%) [Y_0*α*_-Glc-H_2_O-H]^−^, 455.2 (50%) [A-H]^−^	D + H
**M**_25_	C_40_H_62_O_12_	733.4213	14.96	571.4 (100%) [M-Rha (OH)–H]^−^, 553.4 (30%) [M-Rha (OH)–H_2_O–H]^−^, 439.3 (50%) [A-H]^−^, 421.3 (25%) [A-H_2_O–H]^−^423.3 (30%) [A-H_2_O + H]^+^	D + H
**M**_26_	C_46_H_72_O_16_	879.4810	15.01	717.5 (100%) [Y_0*α*_-H]^−^, 571.4 (80%) [Y_0*α*_-Rha-H]^−^, 553, 4 (30%) [Y_0*α*_-Rha-H_2_O-H]^−^, 439.5 (90%) [A-H]^−^423.3 (100%) [A-H_2_O + H]^+^	D
**M**_27_	C_46_H_72_O_17_	895.4766	15.41	733.2 (5%) [Y_0*α*_-H]^−^, 571.4 (60%) [Y_0*α*_-Rha (OH)–H]^−^, 553.3 (30%) [Y_0*α*_-Rha (OH)–H_2_O–H]^−^, 439.3 (100%) [A-H]^−^	D + H
**M**_28_	C_40_H_62_O_11_	717.4270	15.90	571.4 (10%) [M-Rha-H]^−^, 553.4 (10%) [M-Rha-H_2_O-H]^−^, 439.4 (30%) [A-H]^−^423.4 (100%) [A-H_2_O + H]^+^	D
**M**_29_	C_34_H_52_O_7_	571.3714	15.95	553.3 (10%) [M-Rha-H_2_O-H]^−^, 439.3 (60%) [A-H]^−^	D
**M**_30_	C_40_H_62_O_11_	717.4259	16.25	571.4 (10%) [M-Rha-H]^−^, 553.4 (10%) [M-Rha-H_2_O-H]^−^, 439.3 (30%) [A-H]^−^423.3 (20%) [A-H_2_O + H]^+^	D
**M**_31_	C_42_H_64_O_13_	759.4363	16.66	717.5 (25%) [M-Ac-H]^−^, 571.5 (10%) [M-Rha-Ac-H]^−^, 553.2 (20%) [M-Rha-Ac-H_2_O-H]^−^, 439.0 (10%) [A-H]^−^423.4 (75%) [A-H_2_O + H]^+^	D + Ac
**M**_32_	C_29_H_44_O_4_	455.3258	16.91	455.3 (100%) [M-H]^−^439.4 (70%) [A-H_2_O + H]^+^	D + H
**M**_33_	C_34_H_52_O_7_	571.3691	17.15	553.2 (20%) [M-H_2_O–H]^−^, 439.3 (40%) [A-H]^−^	D
**M**_34_	C_34_H_52_O_7_	571.3728	18.61	553.3 (20%) [M-H_2_O–H]^−^, 439.3 (20%) [A-H]^−^	D
**M**_35_	C_30_H_48_O_4_	471.3524	20.31	471.4 (100%) [M-H]^−^455.3 (30%) [A-H_2_O + H]^+^, 437.7 (10%) [A-2 H_2_O + H]^+^	D + H
**M**_36_	C_29_H_44_O_3_	439.3252	21.35	439.3 (100%) [M-H]^−^423.3 (100%) [A-H_2_O + H]^+^	D
**M**_37_	C_29_H_42_O_5_	469.3080	23.65	469.3 (100%) [M-H]^−^453.2 (20%) [A-H_2_O + H]^+^, 435.3 (10%) [A-2 H_2_O + H]^+^	D + O
**M**_38_	C_40_H_62_O_12_	733.4206	9.73	571.4 (100%) [M-Rha (OH)–H]^−^, 553.4 (20%) [M-Rha (OH)–H_2_O–H]^−^, 439.3 (30%) [A-H]^−^	D + H
**M**_39_	C_52_H_82_O_22_	1057.5275	11.31	733.6 (10%) [Y_0*α*_-H]^−^, 587.4 (100%) [Y_0*α*_-Rha-H]^−^	D + H
**M**_40_	C_60_H_94_O_27_	1245.5917	11.45	733.6 (100%) [Y_0*α*_-Ac-H]^−^, 587.4 (20%) [Y_0*α*_-Rha-Ac-H]^−^, 569.4 (10%) [Y_0*α*_-Rha-H_2_O-H]^−^, 455.3 (15%) [A-H]^−^	H + Ac
**M**_41_	C_58_H_92_O_26_	1203.5824	10.11	733.4 (100%) [Y_0*α*_-H]^−^, 587.2 (5%) [Y_0*α*_-Rha-H]^−^, 569.3 (7%) [Y_0*α*_-Rha-H_2_O-H]^−^, 455.2 (10%) [A-H]^−^439.4 (100%) [A-H_2_O + H]^+^	H

A: aglycone; P: prototype; D: deglycosylation; H: hydroxylation; Ac: acetylation; Gu: glucuronidation; G: glycosylation; O: oxidation. Feces: M_0_–M_37_; uurine: M_0_, M_4_, M_7–9_, M_11–13_, M_15_, M_17_, M_20_, M_28_, M_32_, and M_37_–M_40_; plasma: M_0_, M_21_, M_25_, M_28_, M_29_, M_32_–M_36_, and M_41._

## Data Availability

The data used to support the findings of this study are included within the article and the supplementary information files.

## Abbreviations

A: Aglycone
Ara: Arabinose
Glc: Glucose
Rha: Rhamnose
*t*_*R*_: Retention time
SPE: Solid-phase extraction
UPLC: Ultraperformance liquid chromatography
ELSD: Evaporative light-scattering detector.

## Supplementary Materials

Supplementary Materials See Figures S1–S8 in the supplementary material for metabolic pathways and mass spectra of some typical metabolites. See Table S1 in the supplementary material for the ^13^C-NMR data of ciwujianoside B.
