# Supplementary material for: Detection and Characterization of the Metabolites of Ciwujianoside B in Rats Based on UPLC-Fusion Lumos Orbitrap Mass Spectrometry
Source: J Anal Methods Chem. 2024 May 22;2024:3187511. doi: 10.1155/2024/3187511 (PMC11136543; doi:10.1155/2024/3187511)
Supplement: Supplementary Materials — See Figures S1–S8 in the supplementary material for metabolic pathways and mass spectra of some typical metabolites. See Table S1 in the supplementary material for the 13C-NMR data of ciwujianoside B. [file 3187511.f1.docx]

**Figure and table captions**

**Fig. S1**. The ESI^-^-MS spectrum (**A**), ESI^-^-MS/MS spectrum (**B**) of **M_12_** and proposed fragmentation pathway (C).

**Fig. S2**. The ESI^-^-MS spectrum (**A**), ESI^-^-MS/MS spectrum (**B**) of **M_23_** and proposed fragmentation pathway (**C**).

**Fig. S3**. The ESI^-^-MS spectrum (**A**), ESI^-^-MS/MS spectrum (**B**) of **M_37_** and proposed fragmentation pathway (**C**).

**Fig. S4**. The ESI^-^-MS spectrum (**A**), ESI^-^-MS/MS spectrum (**B**) and proposed fragmentation pathway of **M_4_** (**C**). The ESI^-^-MS spectrum (**D**), ESI^-^-MS/MS spectrum (**E**) and proposed fragmentation pathway of **M_6_** (**F**).

**Fig. S5**. The ESI^-^-MS spectrum (**A**), ESI^-^-MS/MS spectrum (**B**) and proposed fragmentation pathway of **M_13_** (**C**). The ESI^-^-MS spectrum (**D**), ESI^-^-MS/MS spectrum (**E**) and proposed fragmentation pathway of **M_40_** (**F**).

**Fig. S6**. The ESI^-^-MS spectrum (**A**), ESI^-^-MS/MS spectrum (**B**) and proposed fragmentation pathway of **M_9_** (**C**). The ESI^-^-MS spectrum (**D**), ESI^-^-MS/MS spectrum (**E**) and proposed fragmentation pathway of **M_11_** (**F**).

**Fig. S7**. The ESI^-^-MS spectrum (**A**), ESI^-^-MS/MS spectrum (**B**) of **M_7_** and proposed fragmentation pathway (**C**).

**Fig. S8**. The numbers of metabolites of ciwujianoside B in feces, urine, and plasma (**A**), the relative percentage of metabolite type(**C**) and distribution of metabolites (**B**) of ciwujianoside B in feces, urine, and plasma.

**Table S1**. ^13^C-NMR data of ciwujianoside B and literature (150 MHz, Pyridine-d 5).

**Fig. S1**

**Fig. S2**

**Fig. S3**.

**Fig. S4**

**Fig. S5**

**Fig. S6**

**Fig. S7**

**Fig. S8**

**Table S1**

| **No.** | Ciwujianoside **B**  *δ*C | Litetature  *δ*C | **No.** | Ciwujianoside **B**  *δ*C | Litetature  *δ*C |
| --- | --- | --- | --- | --- | --- |
| 1 | 38.7 | 38.9 | 3-O-Ara-1 | 104.6 | 104.9 |
| 2 | 26.2 | 26.4 | 2 | 75.6 | 75.9 |
| 3 | 88.5 | 88.8 | 3 | 73.8 | 73.8 |
| 4 | 39.2 | 39.4 | 4 | 68.4 | 68.6 |
| 5 | 55.6 | 55.9 | 5 | 64.4 | 64.5 |
| 6 | 18.3 | 18.4 | Rha-1 | 101.5 | 101.7 |
| 7 | 32.8 | 33.1 | 2 | 72.3 | 72.4 |
| 8 | 39.6 | 39.9 | 3 | 72.5 | 72.6 |
| 9 | 47.7 | 48.0 | 4 | 73.8 | 73.8 |
| 10 | 36.7 | 37.0 | 5 | 69.6 | 69.9 |
| 11 | 23.5 | 23.5 | 6 | 18.3 | 18.6 |
| 12 | 122.9 | 122.6 | 28-O-Glc-1 | 95.5 | 95.8 |
| 13 | 143.2 | 143.4 | 2 | 73.8 | 73.9 |
| 14 | 41.4 | 41.7 | 3 | 78.0 | 78.3 |
| 15 | 27.9 | 28.1 | 4 | 70.0 | 70.3 |
| 16 | 23.5 | 23.5 | 5 | 77.7 | 78.1 |
| 17 | 47.1 | 47.3 | 6 | 69.6 | 69.9 |
| 18 | 47.2 | 47.5 | Glc-1 | 104.6 | 104.8 |
| 19 | 41.8 | 42.1 | 2 | 75.0 | 75.3 |
| 20 | 148.1 | 148.3 | 3 | 76.2 | 76.5 |
| 21 | 29.8 | 30.1 | 4 | 78.4 | 78.7 |
| 22 | 37.4 | 37.8 | 5 | 76.9 | 77.2 |
| 23 | 27.9 | 28.1 | 6 | 61.0 | 61.4 |
| 24 | 16.7 | 17.0 | Rha-1 | 102.4 | 102.7 |
| 25 | 15.4 | 15.6 | 2 | 72.3 | 72.6 |
| 26 | 17.2 | 17.5 | 3 | 72.5 | 72.8 |
| 27 | 25.7 | 26.0 | 4 | 73.8 | 73.9 |
| 28 | 175.5 | 175.7 | 5 | 70.5 | 70.3 |
| 29 | 107.1 | 107.5 | 6 | 18.3 | 18.5 |
